# Supplementary material for: Are carbon emissions trading and green financial instruments synergistic? -Comprehensive quantitative research based on content analysis
Source: PLoS One. 2024 Mar 7;19(3):e0298601. doi: 10.1371/journal.pone.0298601 (PMC10919601; doi:10.1371/journal.pone.0298601)
Supplement: S1 Data — (ZIP) [file pone.0298601.s001.zip › Data/地级市Z_F工作报告.zip!/╣π╢1⁄2/╤⌠╜¡╩╨/└┤╫╘╣1⁄2╓┌║┼í╛┬φ┐╦╩2╛▌═°í┐_2007.docx]

各位代表：

　　我代表市人民政府向大会作政府工作报告，请予审议，并请政协各位委员和其他列席人员提出意见。

过去三年工作回顾

　　本届政府2004年4月产生以来，在省委、省政府和市委的正确领导下，在市人大和市政协的监督支持下，坚持以邓小平理论和"三个代表"重要思想为指导，全面落实科学发展观，调整完善发展思路，以发展为第一要务，以工业为主攻方向，以招商引资为突破口，大力发展临港工业和区域特色经济，着力打造广东电力能源基地和面向西南的物流配送基地，全市国民经济持续快速健康协调发展，社会各项事业全面进步。本届政府确定的各项目标任务顺利完成。

国民经济持续快速增长

　　2006年，预计全市完成生产总值346.7亿元，比上年增长14％，高出预期目标1.5个百分点，三年年均增长13.6%，是我市近10年来经济增长最快的三年；人均生产总值14000元，比上年增长13％，三年年均增长12.4%。城市发展后劲明显增强，跃居全省二类地区首位。全社会固定资产投资101.5亿元，比上年增长21.4％，三年年均增长20.9%；全社会消费品零售总额187亿元，比上年增长17.6%，三年年均增长15.3%；地方财政一般预算收入10.4亿元，比上年增长19.7%，三年年均增长16.5%，全市财政总收入达28.2亿元，收支保持平衡；全市金融机构人民币各项存款余额281.3亿元，比年初增长14.6%，比2003年增长46%；贷款余额106.7亿元，比年初增长13.4%。

产业结构调整成效显著

　　工业主导地位增强。三大产业比例由2003年的34∶31.8∶34.2调整为2006年的24.4∶39.6∶36，第二产业的比重全面超过第一、三产业。电力能源和临港工业发展势头良好，阳江核电站、阳西燃煤电厂正式动工建设且进展顺利，阳江抽水蓄能电站和海陵风力发电场进入可研阶段；丰源粮油临港加工区、川崎特殊钢有限公司等先后建成投产，德恩富光盘生产基地、龙尚鞋业等项目正在建设。传统优势行业创新能力不断增强，培育发展了 "十八子"、 "宝马利"、"新力"等一批高新技术企业和民营科技企业，产业结构进一步优化升级。工业园区建设加快。中山火炬（阳西）、中山石歧（阳江）、佛山禅城（阳东）3个省级产业转移工业园和高新技术产业开发区、阳东经济开发区等工业园区建设势头良好，基础设施日益完善。2006年，全市工业总产值达372.9亿元，比上年增长15%，三年年均增长18.6%；工业增加值104.6亿元，比上年增长20%，三年年均增长23%。

　　农业基础地位巩固。粮食综合生产能力有效提高，创建了共15万亩的十大粮食生产基地。农业产业结构进一步调整优化，优势经济作物和特色农业加快发展。农业产业化进程稳步推进。至2006年底，全市培育了省级农业龙头企业3家、市级农业龙头企业15家、农业专业化合作组织107个，带动农户26.3万户，占农户总数的56%，户均增收1816元。林业发展加快，全市建成商品林基地255万亩，阳春市、阳西县成功创建了广东省林业生态县。畜牧业进一步发展，三年引进畜牧业企业13家；高致病性禽流感等重大动物疫病得到有效防控。2006年，全市农业总产值137.9亿元，比上年增长5.5%，三年年均增长6.2%。

　　第三产业发展加快。信息产业、社会服务等新兴服务业迅速崛起，住房、汽车、通信等消费快速增长，成为新的经济增长点。专业市场日趋完善，全市新增5000平方米以上专业批发市场5个。连锁经营企业不断增多，国美电器等现代商贸流通企业落户阳江。市区私人自建住宅管理不断加强，房地产业快速健康发展，三年来，全市商品房销售面积和销售额分别年均增长9.1%和38.9%。2006年，全市第三产业实现增加值124.7亿元，比上年增长14.2%，三年年均增长16.3%。

社会主义新农村建设扎实推进

　　农村规划建设不断加强，推广使用《阳江市城镇住宅设计图集》，20个新农村建设联系点工程全面铺开，村镇住宅建设水平进一步提高。农村人居环境日益改善，镇通建制村公路硬化建设走在全省前列，三年共完成镇通建制村公路硬化建设1682公里，惠及512个行政村160万人口；建设农村饮水工程32宗，受益人口32.8万；改造贫困农户泥砖住房5.3万户400.3万平方米，累计完成需改造泥砖房总面积的89%。农田水利建设进一步加强，投入资金3.8亿元，加固江海堤围57公里、中小型水库51座，清淤灌区干支渠1860公里。农村税费改革稳步推进，三年来，每年减免农民负担2.3亿元，农民年人均减负141.7元；2005年全面免征农业税，全市农民实现零税负。农村劳动者的技能培训逐步加强，农村富余劳动力转移就业30多万人。农村各项社会事业不断发展，新型农村合作医疗覆盖面由2003年的28%提高到2006年的64%；全面免除农村户籍学生义务教育阶段学杂费。农村各项管理进一步规范，村务公开工作不断加强，阳西县荣获"全国村务公开民主管理示范单位"称号；全市704个村委会均实行村级财务公开，逐步建立起一套行之有效的村委会经济活动监督机制。创建生态文明村活动深入开展，农村文明程度不断提高。

特色经济发展活力增强

　　旅游业有新成效。开发建设了涛景度假村、东方银滩欢乐广场、阳西咸水矿温泉等景区景点，三山岛国际旅游度假区和碧桂园凤凰酒店等5家五星级酒店建设进展顺利。海陵岛入选"全国十大最美海岛"，阳春凌霄岩被评为国家地质公园，阳江温泉度假村被评为五星级酒店。2006年，全市接待游客471.7万人次，三年年均增加64.7万人次；旅游总收入24.8亿元，三年年均增加3.8亿元。

　　海洋渔业有新进步。2006年，全市水产品总产量93万吨，渔业总产值54.2亿元，分别比2003年增长7.7%和24.9%。海捕、海养和水产品总产量、人均产量等指标一直保持全省地级市首位。水产品加工业提速发展，万事达海洋食品有限公司、顺兴海洋渔业有限公司等加工企业建成投产，谊林海达速冻水产有限公司发展到年产值6亿元，年出口额达5761万美元，居全省同类企业首位。渔港建设不断加强。2004年以来，投入渔港建设资金3649万元，渔港基础设施进一步完善，东平、沙扒渔港被评为全国一级渔港。渔民上岸安居工程建设成效显著，多次受到省的表彰。

　　南药生产加工有新发展。建成了春砂仁、益智、沙姜、藿香、淮山、胡椒、金钱草、穿心莲、凉粉草等九大南药生产基地，2006年，全市南药种植面积14.1万亩，比2003年增长166%，年均增长55.3%；南药加工业迅速发展，引进了一片天制药有限公司等一批医药企业，南药加工企业由2003年的10家增加到15家，加工能力不断增强。2006年，南药总产值4亿元，比2003年增长167%，年均增长55.7%。

内外源型经济协调发展

　　招商引资势头强劲。制定并实施了《关于进一步加强招商引资工作的决定》、《阳江市招商引资奖励办法》、《阳江市降低收费门槛，促进招商引资的决定》等政策措施，招商引资主体下移到县镇，各级招商引资积极性有效提高。在美国、澳大利亚及香港等地设立招商引资联络处，聘请招商顾问或代理人，招商效果进一步提高。三年来，先后引进了美国嘉吉集团、广东珠江投资集团、日本山光社、澳门吴福集团等一批国内外大企业到我市投资。合成油、生物柴油、金光林板浆纸、阳江棉花储备中心等一批重大项目正在洽谈。2004年以来，全市累计签订各类合作项目100多项，合同金额8.22亿美元；实际利用外商直接投资2.39亿美元，年均增长61.8%，其中2006年实际利用外商直接投资1.06亿美元，比上年增长40.4%，是建市以来实际利用外商直接投资数额最大的一年，受到省的表彰和奖励。

　　对外贸易持续增长。至2006年，全市外贸出口总额10.1亿美元，比上年增长10%，三年年均增长12%。出口产品达400多种，遍及日本、欧美、港澳台等国家和地区，出口产品结构和市场结构进一步优化。进口工作得到加强。2006年，全市进口总额1.5亿美元，比上年增长40%，三年年均增长8.9 %。

　　民营经济快速发展。2006年底，全市民营企业与个体工商户44833户，比2003年净增6686户。2006年民营经济实现增加值203.8亿元，比上年增长14.2%，拉动全市经济增长8.4个百分点。其中民营工业企业和个体工业户6978家，实现产值282.5亿元，占全市工业总产值75.8 %。全市年产值超亿元民营工业企业累计达到20家，比2003年增加10家；民营企业出口总额5亿美元，占全市出口总额的52%。民营企业逐步向科技型、工贸一体化和自营进出口方向发展。

各项改革不断深化

　　行政体制改革有序推进，综合设置了文化广电新闻出版局，单独设置了安监局和市国有资产监督管理委员会，调整了市发改局、经贸局、农业局、卫生局的部分职能，撤并了9个镇，行政效率进一步提高。事业单位改革稳步推进，完成了市建筑设计院等4家市直勘察设计单位的改革。行政审批制度改革取得新成效。2004年以来，全市共取消、减少行政许可事项190项，清理规范性文件和政策措施315份。国有企业改革向纵深推进。引进了广东粤电集团参与阳江港改制重组，阳江港开发总公司等14家企业的改制工作顺利完成。农村信用社改革取得较好成绩。国土资源管理体制改革圆满完成，管理工作进一步加强。粮食流通体制改革不断深化，国有粮食企业改革取得新突破。投融资、财政监管、水利工程管理等体制改革稳步推进。

发展环境日益优化

　　基础设施建设取得新突破。交通网络建设进一步完善，阳茂高速公路阳江段、S113线阳春南段和S369线阳春段一期工程、程梁公路、阳江港3万吨级粮食码头和5万吨级航道疏浚工程等先后建成并投入使用，S113线阳春南段和S369线阳春段二期工程正在建设，开阳高速公路阳江市区立交、阳茂高速公路阳西县城连接线和阳江港至云浮高速公路建设前期工作进展顺利。城乡电网建设进一步完善，新建了6个110千伏和漠南220千伏变电站，扩建了阳江220千伏变电站，电网"卡脖子"问题得到有效解决。城市规划、建设和管理水平进一步提高，西平北路、体育北路、安宁中路等一批城市主干道和图书馆、科技馆、博物馆、群众艺术馆、文化广场等文化设施相继建成；市自来水厂三期和市第一净水厂二期扩建工程建设加快；引进了广东碧桂园集团参与城市开发建设，阳东燕山湖综合开发项目建设进展顺利；城南新区开发建设全面展开，江朗大道、富康路、东门南路、二环南路等主干道路正在建设；城镇化水平不断提高，中心镇辐射带动作用日益增强；城市管理不断加强，市容市貌进一步改善。城乡水利防灾减灾工程建设稳步推进，完成了市区防洪示范工程和阳春城区防洪工程部分堤段的建设，"三防"基础设施不断完善。

　　投资软环境不断改善。坚持每年开展一次整顿机关作风大行动和行风评议活动，开办了《行风建设大家谈》广播电视节目，强化行风监督，全市机关作风明显好转。各级投资服务管理中心建设不断加强，投资服务体系日益健全，服务水平、办事效率明显提高。大力开展清费减负，营商成本进一步降低。2004年以来，全市取消行政事业性收费141项，减免涉工规费41项，每年减少行政事业和经营服务性收费1900多万元。

社会各项事业全面进步

　　科技创新能力有新提升。科技创新体系日益完善，新增2家省级工程技术研发中心、1家省级企业技术中心、1家省级农业科技创新中心和2个省级技术创新专业镇；建立了省级火炬计划特色产业基地、五金刀具产业集群升级示范区和市五金刀剪技术创新中心；新增2家国家级和40家省级高新技术企业、24家省级民营科技企业。产学研合作不断加强，建立完善了华南理工大学阳江工业技术研究院，成功举办了大型科技成果推介会，三年来共实施6项国家级、86项省级科技计划项目。科技进步对经济增长的贡献率比2003年提高1.5个百分点。知识产权保护工作继续加强，建立了全国首个五金刀剪专利信息数据库，全市专利申请量和授权量在全省排第11位和第12位，连续多年保持粤西地区领先地位，阳江被批准为"广东省知识产权试点城市"。三年来，全市新增中国驰名商标1件、中国名牌产品1个、国家免检产品4个、原产地域保护产品2个、商务部重点培育和发展的出口品牌1个、广东省著名商标20件、广东省名牌产品16个、广东省高新技术产品16个。

　　教育事业迈出新步伐。三年来，全市投入教育资金5.3亿元，新建、扩建、改造学校300多所， 14所学校晋升为省一级学校。基础教育管理体制不断完善，全面实施义务教育"一费制"， "普九"教育得到巩固提高。普通高中教育发展加快，新建了市同心中学和阳东广雅中学，三年增加高中学位1.8万个。2006年全市每万人口普通高中在校生179人，在全省排名第14位。阳江一中、阳春一中顺利通过了广东省国家级示范性普通高中初期督导验收。中等职业技术教育加快发展，市技工学校新校区建成招生，并晋升为省一类技工学校，在校生由原来的500多人增加到3000多人。高等教育稳步发展，阳江职业技术学院办学规模逐年扩大，阳江广播电视大学通过省办学条件现代化标准验收。教育质量稳步提高，2006年高考全市每万人口上重点线人数在全省排名第16位。

　　文化、卫生等各项事业取得新发展。广东海上丝绸之路博物馆建设顺利推进，群众文化活动丰富多彩，文艺创作日益繁荣，阳江被授予"中国诗词之市"、"中国楹联文化城市"和"广东省音乐之乡"称号。人才工作继续加强。三年来，全市共组织各种大型人才招聘会9场，为企业引进各类人才近万名。公共卫生三大体系建设不断推进，突发性公共卫生事件预警和应急机制日益完善，医疗救治能力明显增强。无偿献血工作连续三次获全国先进城市称号。体育事业不断发展，广泛开展群众性体育运动，成功承办了世界华人风筝赛、全国男子柔道冠军赛和第十二届省运会帆板赛等多项大型体育赛事，组团参加第十二届省运会取得了历史最好成绩，金牌总数超过建市以来参加省运会取得的金牌总数。人口和计划生育工作继续加强，在全国率先实行节育奖，人口自然增长率控制在7‰以下，出生人口性别比偏高问题得到有效遏制，连续多年受到省政府的表彰。环境友好型社会建设不断推进，环境保护责任制考核连续三年被省评为优秀。妇女儿童发展规划有效实施。依法管理民族宗教事务水平提高。爱国卫生运动广泛开展，连续两次获得"广东省卫生城市"称号。国防动员、兵员征集、民兵预备役和双拥工作进一步加强，首次获得"国家双拥模范城"和第四次获得"广东省双拥模范城"称号。殡改工作有效推进，在全省排名由2003年的第11位跃升为第7位。档案工作的存史、资政、利民作用进一步增强。外事、侨务、民政、统计、人防、打私、气象、地震、市志等各项工作取得新成绩。

和谐阳江建设初见成效

　　就业和社会保障工作不断加强。三年来，全市新增就业岗位9.6万个，国有企业下岗失业人员再就业2.5万人，城镇登记失业率控制在3.2%以下。城乡社会保障体系不断完善，全市养老、失业、医疗、工伤和生育保险参保人数达71.2万人，年均增长9.3%；获得最低生活保障的城乡居民8.5万人，比2003年增加4.9万人。成立了市慈善会，募集资金700多万元。救助特困人口32.5万人次，发放临时救助金1825万元。

　　群众生活环境逐步改善。三年来，在市区建设经济适用住房和廉租住房574套4.3万平方米，缓解了市区困难群众住房难问题；成立了10个法律援助中心，为特困、特残群众提供法律援助。全面开放市区五大公园，免费让市民群众入园娱乐。完成了清理拖欠农民工工资和建设领域拖欠工程款工作。全力做好抗洪救灾复产工作，在台风"派比安"正面袭击阳江后，组织动员全市干部群众奋起抗灾，确保了受灾群众有饭吃、有水喝、有衣穿、有地方住、有病能及时治疗，没有一个受灾群众子女因灾辍学，全市3036户"全倒户"2007年元旦前全部住上新房，取得了救灾复产的全面胜利。群众生活质量有了较大提高，2006年，全市城镇居民人均可支配收入10079元，年均增长8%；农民年人均纯收入4586元，年均增长5.9％，入选"2006中国城市生活质量50佳"城市，居全省地级市第八位。

　　社会保持和谐稳定。强化公安队伍和社会治安防控网络建设，开展声势浩大的打击"双抢"、"两盗"等多发性犯罪，严厉打击各种刑事犯罪，社会治安状况明显好转，群众安全感明显增强。消防工作成绩突出，市政府被授予"2002-2005年度全省消防工作先进单位"荣誉称号。加强信访工作，坚持领导接访和领导包案调处制度，妥善处置群体性事件，及时化解各类社会矛盾，集体上访人数逐年下降。深入开展安全生产专项整治，全市安全生产形势稳定。2004年以来，全市工矿商贸企业未发生特大安全事故，一般安全事故大幅下降。整顿和规范市场经济秩序取得阶段性成果，偷税骗税、非法传销、制售假冒伪劣商品等违法犯罪活动得到遏制，食品药品专项整治成效明显。扎实开展保持共产党员先进性教育活动，理想信念和职业道德教育不断加强。群众性精神文明创建活动深入开展，涌现出一批文明窗口、文明村镇和文明社区。阳江连续两次被授予"广东省文明城市"称号。

民主法制和政府自身建设得到加强

　　坚持和完善向人大及其常委会报告工作、向政协通报情况制度，自觉接受监督。三年来，市政府共办理人大议案6件、代表建议341件、政协建议案3件、政协委员提案346件，办复率均达100 %。加强与民主党派、工商联、无党派人士和工、青、妇等人民团体的联系，民主渠道进一步拓宽。政务公开纳入法制化轨道，企事业单位民主管理制度不断健全。圆满完成了"四五"普法工作任务，启动了"五五"普法工作，人民群众法律意识增强。不断加强政府自身建设，政府职能进一步转变。认真贯彻落实《公务员法》、《行政许可法》等法律法规，制定和修订了《阳江市人民政府工作规则》、《阳江市实施行政许可规定》等规范性文件，政府依法行政水平不断提高。强化法制、审计、监察工作，严肃查处违法违纪案件和纠正违规行为，廉政建设得到加强。

　　各位代表，过去的三年，是我市经济和社会发展取得显著成绩的三年。这些成绩的取得，是省委、省政府和市委正确领导的结果，是各级人大及其常委会、人民政协和各民主党派监督支持的结果，是全市人民同心同德、奋力拼搏的结果。在此，我代表市人民政府向全市广大工人、农民、知识分子、干部职工、驻阳江人民解放军、武警官兵、人民警察和各界人士表示崇高的敬意！向关心和支持阳江建设的港澳台同胞、海外侨胞和国际友人表示衷心的感谢！

　　回顾三年来的工作，我们深深地体会到，要开创我市现代化建设新局面，必须坚持以邓小平理论和"三个代表"重要思想为指导，牢固树立和落实科学发展观，推动经济社会又好又快发展；必须把做大经济总量作为经济社会发展的中心工作，创新发展思路，加快发展速度，提高发展质量；必须坚持招商引资与资源节约、环境保护并举，既大力招商引资，又不能患投资饥饿症，走高污染、高耗能、低效益的老路；必须统筹城乡协调发展，既大力发展工业，又狠抓农业农村经济，让社会各阶层共享改革发展成果；必须坚持以人为本，着力帮助群众解决生产生活困难，切实维护群众切身利益，推进和谐阳江建设。

　　我们也清醒地看到，我市经济社会发展还存在不少困难和问题。主要是：经济总量小，结构不合理；固定资产投资规模小，经济增长短期内动力不足；科技创新能力弱，产业竞争力不强；教育事业发展相对滞后，高素质人才缺乏；新增税源不足，财政增长乏力；农村发展、农民增收仍存在瓶颈，部分群众生活还比较困难；整治社会治安和市场经济秩序任务依然艰巨，医疗卫生、安全生产和食品药品安全等方面还存在薄弱环节等。对这些问题，我们将认真采取措施加以解决。

今后五年奋斗目标和2007年工作意见

　　今后五年，是我市努力实现跨越发展、追赶全省发展水平的关键时期。目前，国际、国内区域合作不断扩大，产业资本加速向低成本地区转移。国家和省对欠发达地区实施积极的发展扶持政策。我市经过19年的发展，积聚了一定的发展基础，经济社会进入了新一轮快速增长期。这些都有利于我市进一步加快发展。但是我市发展中的一些深层次矛盾和问题还未能从根本上得到解决，增大了加快发展的压力。为此，我们必须认清形势，抢抓机遇，发挥优势，创新发展，推动经济社会发展实现新的跨越。

　　今后五年政府工作总的指导思想是：高举邓小平理论和"三个代表"重要思想伟大旗帜，认真贯彻中央和省的决策精神，按照市第五次党代会提出的战略部署，以科学发展观统领经济社会发展全局，以建设现代化阳江为总目标，实施工业立市、旅游强市、商贸旺市、农业稳市、科教兴市战略，推动阳江跨越发展，推进社会事业、文化名城、民主法治、社会主义新农村、绿色阳江建设，加快构建社会主义和谐社会，为与全省同步全面建成小康社会、率先基本实现社会主义现代化打下更为坚实的基础。

　　今后五年的奋斗目标：一是综合实力增强。全市生产总值突破600亿元，年均增长12%以上。产业结构进一步优化，经济增长的质量和效益明显提升，三大产业比例调整为19∶44∶37。二是创新能力提升。科技创新体系进一步健全，产学研结合更加紧密，科研成果有效转化，先进适用技术和高新技术广泛应用，高新技术企业加快发展，科技进步对经济增长的贡献率达50%以上。三是城乡协调发展。城乡规划建设和管理进一步加强，农村社会事业加快发展，社会主义新农村建设初见成效，逐步形成功能互补、布局合理、协调互动的新型城乡关系，城镇化水平达50%以上。四是生态环境优美。循环经济、清洁生产和清洁能源推广取得新成效，环境保护和生态建设不断加强，人与自然和谐发展，单位生产总值能耗下降18%，主要污染物排放总量减少10%，环境综合指标保持优秀等级。五是人民生活改善。城乡居民生活水平和生活质量明显提高，价格总水平基本稳定，全市城镇居民人均可支配收入年均增长7%，农村居民人均纯收入年均增长6%。六是社会和谐稳定。基本形成法治、诚信、公平、文明的社会环境。全市城镇登记失业率控制在3.5%以下，社会保障体系进一步健全，全市参加基本养老保险人数达到33万人，参加基本医疗保险人数达到26万人，新型农村合作医疗保险覆盖面达到95%以上，人民群众切身利益得到有效解决。

　　2007年是新一届政府的开局之年。今年全市经济社会发展的主要预期目标是：生产总值增长13%，单位生产总值能耗下降3.5%，工业总产值增长20%，农业总产值增长5.5%，社会消费品零售总额增长14%，固定资产投资增长20%，外贸出口增长10%，实际利用外商直接投资增长20%，居民消费价格总水平涨幅控制在3%左右，地方财政一般预算收入增长12%，城镇登记失业率控制在3.5%以内，人口自然增长率控制在7.4‰以下，全市城镇居民人均可支配收入增长9%，农村居民人均纯收入实际增长5.5%。

　　按照上述要求，实现全年目标，必须着力做好以下工作：

　　一、强力推进工业发展，壮大工业规模

　　推进一批电力能源项目建设。加快阳江核电站、阳西燃煤电厂建设步伐，推进阳江抽水蓄能电站和海陵、阳东风力发电场前期工作，抓好广东大唐国际粤西能源项目跟踪落实，积极争取国家和省布点我市建设新的重大能源项目，加快电力能源基地建设。

　　上马一批临港工业项目。加快洛玻阳江基地、德恩富光盘生产基地、龙尚鞋业的建设，抓好电子、合成油、生物柴油、金光林板浆纸等重大项目的落实，力促61个在建制造业项目建成投产，推动形成以重化工业和装备制造业为主的临港工业产业集群。

　　打造一批吸引力强的工业园区。全面推进高新区、阳东经济开发区、环保工业园和中山火炬（阳西）、中山石歧（阳江）、佛山禅城（阳东）、广州花都（江城）、东莞长安（阳春）等5个产业转移园区等各类工业园区开发建设，进一步完善工业园区的路、水、电、通讯等基础设施，吸引项目集聚，推动工业跨越发展。

　　提升一批传统优势企业。广泛运用信息、节能、新型环保等高新技术和先进适用技术改造提升传统产业，重点抓好阳春钢铁公司的技术改造，扶持一批具有自主知识产权和品牌、主业突出、核心竞争力强的企业做大做强，培育支柱产业。

　　二、大力开展招商引资，推动内外源型经济互动发展

　　进一步优化投资软环境。减少审批事项，简化审批手续，严格实施首问责任制、工作承诺制和行政问责制，营造规范、便捷、优质、高效的服务环境。完善和落实鼓励投资发展的政策措施，营造优惠、互利、宽松、平等的政策环境。继续开展清费减负工作，降低收费门槛，营造收费规范、成本低廉的营商环境。加大依法治市的力度，提高全社会的法律意识和依法办事水平，营造严格执法、公正司法的法制环境。把阳江打造成承接珠三角和港澳台等地区新一轮产业转移最具吸引力的城市。

　　努力扩大招商引资规模。完善招商引资激励机制，重奖招商引资有功人员，调动全社会招商引资的积极性。大力推行项目招商、委托招商、代理招商，提高招商引资成效。进一步密切与珠三角等发达地区的联系，推进产业转移园区共建工作，更加有效地承接发达地区的产业转移。加强对欧美、日本等发达国家和地区的宣传推介，创造条件引进跨国公司特别是世界500强企业来我市投资设厂。充分利用临港优势和资源优势，引导外资投向高新技术、石油化工、装备制造和资源加工等产业。认真筹办第六届刀剪博览会和第七届旅游文化节，积极组织企业参加"泛珠三角"各种经贸活动。加强对已经签约项目的跟踪和协调服务，提高履约率。

　　促进外贸出口有效增长。鼓励具有自主知识产权和自有品牌产品扩大出口，扶持农产品深加工出口，提升五金刀剪、家具等传统产品出口档次。大力发展一般贸易和加工贸易，推进发展技术贸易和服务贸易。赋予更多符合条件的民营企业进出口经营权，加大中东、非洲、南美、印度和俄罗斯等新市场的开拓力度，巩固欧盟市场，推动出口主体和市场多元化。继续组织企业到国外重点市场举办经贸洽谈活动，扩大国外重点市场出口。完善外贸公共服务体系，建立重点出口商品反倾销分析预警机制，提高企业应对贸易壁垒能力。

　　推动民营经济发展提高。建立完善中小企业服务体系，进一步帮助民营企业解决融资难等实际问题。鼓励推动有资金、有实力、有市场的民营企业开展低成本扩张，通过兼并、收购、联合等形式做大做强。支持有条件的企业"走出去"，开拓国内外市场。鼓励和引导民营企业加强体制和科技创新，提高自主创新能力，创立自主知识产权，创建名牌产品，提高民营经济发展水平，促进内源型经济加快发展。

　　三、着力繁荣农业和农村经济，加快社会主义新农村建设

　　大力发展现代农业。落实扶持粮食生产政策，稳定粮食生产能力。加大农业结构调整力度，大力发展优势经济作物和特色畜牧业。抓好红丰、平地等省级农业现代化示范区建设，培育发展特色农产品产业带。扶持农业龙头企业，培育农民专业合作组织，提高农业生产专业化和社会化程度，推进农业产业化经营。重点扶持3家省级、15家市级农业龙头企业发展，增强带动农户的能力。加强农业标准化工作，健全农产品质量安全监测体系，打造优质安全的农产品区域品牌。加大农业科技推广力度，加快农业机械化进程，提高农业科技含量。加强动植物疫病防控，保障农业生产安全。

　　加快推进新农村建设。坚持城乡规划一体化，加强农村规划、建设和管理，重点推进阳东县北惯镇平地村村镇规划试点工作，抓好20个新农村建设联系点。扩大公共财政覆盖农村的范围，增加农村社会公共事业方面的投入，改善农村公共服务。积极推进网络到镇、信息进村工程，完善农村道路、电网、通信等基础设施。深入开展创建生态文明镇村活动，大力推广应用沼气，改善农村生态环境。加强农村文化建设，深入开展群众性精神文明创建活动，提高农村文明程度。

　　着力提高农民收入。巩固农村税费改革成果，加强减轻农民负担工作的监管，严防农民负担反弹。完善农业补贴政策和最低收购价制度，保障农民务农收益。有针对性地进行实用技术和岗位技能培训，增强农民致富本领。有序引导农村富余劳动力向二、三产业转移、农村人口向城镇集聚，增加农民非农收入。

　　四、大力发展区域特色经济，做大做强县域经济

　　加快发展旅游业。抓好旅游规划、资源整合与开发，加大旅游宣传推广力度，培育五大旅游板块。一是以涛景度假村为龙头，加快东湖星岛旅游区的开发，完善阳江温泉度假村配套设施，打造东部健康娱乐、休闲度假旅游板块；二是以市区五大公园为龙头，加快东方海湾大酒店、碧桂园凤凰酒店、利福泰大酒店等五星级酒店和世纪公园、碑林等的建设，打造中部商贸娱乐、城市风情旅游板块；三是以广东海上丝绸之路博物馆为龙头，加快三山岛国际综合旅游度假区、马尾岛国际休闲旅游度假区等的建设，打造南部历史文化、滨海休闲旅游板块；四是以凌霄岩国家地质公园为龙头，加快鹅凰嶂风景区等旅游资源的开发，打造北部奇峰异洞、乡村农家游、温泉养生旅游板块。五是以阳西东湖咸水矿温泉度假山庄为龙头，加快东方月亮湾旅游度假区、大垌山旅游风景区等的建设，打造西部医疗保健、生态休闲旅游板块。加强旅游文化建设，以刀剪、风筝、漆器、根雕、奇石、书画等为切入点，推进具有地方文化特色的旅游商品、纪念品开发，促进阳江民俗文化与旅游的融合，提高旅游文化品位。

　　优化发展海洋产业。大力发展水产养殖业，重点推进对虾、鳗鱼、罗非鱼和牡蛎等养殖示范基地建设，培育沿海养殖产业带。加强远洋捕捞基地建设，巩固提高远洋捕捞业。突出抓好水产品深加工，加强水产品产地认证，培育水产品品牌，提高水产品附加值。加强水产品市场体系建设，搞活水产品流通。抓好全国渔业科技入户示范工程阳西县试点和阳西、阳东水生动物疫病防治站两个农业部建设项目，健全水产技术推广体系和水生动物防疫检疫体系。加强渔港建设，推进东平渔港创建国家中心渔港，加快渔民上岸安居工程建设步伐。强化海洋与渔业综合管理，抓好3个国家级海域使用管理示范县建设，创建南鹏列岛省级水产自然保护区，促进海洋和渔业资源可持续发展。办好第五届南海（阳江）开渔节。

　　推进南药产业化经营。继续抓好南药九大生产基地建设，推广南药规范化种植，提高南药生产规模和质量，力争年内种植面积达到20万亩。推进阳江药材交易市场、阳春中医药物流中心建设，搞活南药流通。继续推进一片天制药有限公司产学研基地建设，加强与广州中医药大学等科研院校合作，提升南药研发能力，推动南药加工业加快发展。

　　五、提高自主创新能力，建设创新型和环境友好型社会

　　加快自主创新步伐。积极实施自主创新"五大工程"，强化企业自主创新主体地位，培育发展创新型中小企业。扶持企业创建研发机构，发展一批省级和国家级工程技术研发中心。推动产学研合作，鼓励支持高校和科研院所在我市企业建立研发中心和创新基地，围绕我市支柱产业开展科技攻关。充分发挥华南理工大学阳江工业技术研究院的作用，完善市五金刀剪技术创新平台，组建农业技术创新平台，建立行业共性技术研发中心，推广应用高新技术和先进适用技术改造提升传统产业，培育发展高新技术产业。创建国家级五金刀具和省级水产养殖火炬计划特色产业基地。加强专业镇建设，提升专业镇产业层次和技术水平。全面实施知识产权试点城市工作计划，加强知识产权保护，推动专利技术的开发应用和科研成果转化。深入实施名牌带动战略，推动产业集群和创建区域品牌。进一步深化科技体制改革，完善科技成果评价和科研激励机制，引导社会资金和科技资源投入自主创新。加强科普工作，推广科学技术，提高全民科技文化素质。

　　加强生态环境保护。实施环境规划，推行建设项目环保准入，严格执行省委张德江书记关于环境保护的"三句硬话"：新建项目，凡不符合环保要求的，一律不准上马；在建项目，凡环保不达标的，一律不准投产；已建项目，凡经过限期治理和改造仍不达标的，一律关闭。继续实施治污保洁工程，加强漠阳江、那龙河等流域的综合整治，搬迁沿江两岸污染企业，加强机动车尾气污染防治。加快市环境监控中心建设，建立健全重大环境事件应急监控和预警体系，加强环保执法和应急处理，维护环境安全。加快生态公益林体系建设，强化林地、野生动植物、湿地资源和沿海防护林带的保护管理。重视生态环境保护，构筑绿色生态屏障，保护阳江的青山绿水、碧海蓝天。

　　推进资源节约。实施土地利用总体规划，科学配置土地资源，节约集约利用土地。综合利用新技术、新工艺和新设备，推进钢铁、五金、建材等重点行业、企业节能降耗。积极应用新型墙体材料，大力推广节能建筑。建立统一协调的水资源管理体制，促进节约用水。建立节约型社会评价指标体系，推进节约型企业、社区、学校、机关等的建设，加快建设节约型社会。

　　六、加快基础设施建设，保持投资稳定有效增长

　　完善功能性基础设施。积极推进阳江港至云浮高速公路建设的前期工作，力争年内动工建设；加快S113线阳春南段和S369线阳春段二期工程建设步伐，推动开阳高速阳江市区立交及连接线、阳茂高速织篢立交及连接线、开阳高速公路那龙出口至核电站公路、沿海高速公路延长线白沙至程村公路年内动工建设。全面推进蝶岭500千伏，春城、惯平220千伏和塘坪、长兴、新吉110千伏等6个输变电站的新建扩建工程建设。抓好城乡水利防灾减灾工程建设和基本农田灌区改造。

　　加强城镇建设。继续实施 "北优东联、南拓西进、拥江向海"的城市发展规划。加快江朗大道、富康路、东门南路、二环南路建设，全面推进城南新区开发，打造集行政、商住、文化、休闲为一体的生态型现代新城区。以推进共青湖核电科研后勤生活基地建设为切入点，优化城区北部规划建设，打造城市新亮点。完善城镇配套设施，加快市区马曹路、文明路、创业路等城市主干道建设，推进奕垌垃圾场二期工程和中心镇污水处理厂建设，完成市自来水厂三期和市第一净水厂二期扩建工程建设。配合城市防洪工程建设，推进"一河两岸"的规划整治。加强城市综合管理，强化交通秩序和"六乱"整治，努力营造安全、有序、文明、整洁的城市环境，提升城市形象。创新小城镇建设模式，增强中心镇辐射带动作用，加快城镇化步伐。认真落实国家房地产宏观调控政策，完善房地产市场体系，推动房地产业健康有序发展。

　　打造面向西南的物流基地。认真抓好阳江保税物流中心、国家棉粮油储备基地、粤西烟草配送中心、粤西粮油批发配送中心和阳江五金刀剪商贸城等项目的落实和建设。加快推进3.5万吨级通用码头、生物柴油码头、阳江港疏港大道、阳阳铁路码头专用线和口岸综合服务大楼等的建设。进一步做好10万吨级深水码头建设前期工作，完善港口配套设施。巩固提高港口定期货物航班的运输服务水平，拓展港口物流配送功能，打造面向西南的区域性物流基地。

　　七、深化各项改革，推动体制机制创新

　　推进国有企业改革。以产权制度改革为重点，大力发展以股份制经济为主要形式的混合所有制经济。盘活国有企业闲置资产，多渠道、多形式解决改革资金，妥善安置分流职工。严格执行省委张德江书记关于国企改革的"三条准则"：改革、改制方案未经职代会或职工大会审议通过的，不能实施；职工安置、经济补偿、社会保险接续等措施不落实的，不能实施；职工安置方案和社会保障办法不明确、资金不落实的，不得进入破产程序。完善国有资产监管体制，规范国有资产交易行为，防止国有资产流失。

　　深化投融资体制改革。放宽民间投资领域，引导鼓励民间资本以独资、合资、合作、联营、特许经营等方式参与市政、道路、港口等基础设施项目的建设和经营。加强与国家开发银行的合作，积极利用国家开发性贷款，推进我市基础设施和公共事业项目建设。完善政府投资体制，探索和推行行政公益性项目代建制。深化农信社体制改革，建立稳定的金融协调机制。

　　完善公共财政体制。强化国库集中支付、政府采购制度和"收支两条线"管理，深化部门预算，探索建立财政支出绩效评价体系，提高财政资金使用效益。进一步完善市对县（市、区）的激励型财政管理体制，建立激励型财政转移支付制度。理顺跨县区税收征管关系，强化税收征管，堵塞税收征管漏洞。加大税法宣传，建立良好治税环境。

　　规范市场经济秩序。严厉打击制假售假、走私贩私等各种违法犯罪活动。坚决打击欺行霸市、强买强卖、逃废债务等不法行为。强化产品质量监督，深化食品药品专项整治。加强社会信用体系和机制建设，营造讲求商业道德、诚实守信、公平竞争氛围。

　　八、全面发展各项社会事业，促进经济社会协调发展

　　大力发展教育事业。坚持把教育摆在优先发展的地位。规范发展学前教育。巩固提高义务教育，进一步调整农村中小学布局，全面完成农村义务教育阶段学校危房改造任务，稳步推进义务教育规范化学校建设；按照省的部署，从2007年秋季起，免除全市农村义务教育课本费。加快发展普通高中教育，重点抓好阳春一中等11所中学的迁扩建工作，进一步解决高中学位不足问题；加快阳江一中、阳春一中国家级示范性普通高中建设步伐，力争年内通过评估验收。大力发展中等职业教育，在推进市技工学校二期工程建设的同时，力争各县（市、区）都建设一所中等职业技术学校。办好高等教育，完善阳江职业技术学院校区建设和办学条件，力争通过国家人才培养水平评估验收。加快教育信息化进程，年内实现全市中小学全部接入教育城域网。加强教师队伍建设，提高教学水平和教育质量。

　　全面发展文化、体育、卫生等社会事业。以实施"南海一号"水下考古项目为契机，加强文物、珍贵档案和非物质文化遗产保护。完成市图书馆、博物馆、科技馆和群众艺术馆建设的扫尾工作，完善文化基础设施。加强文学艺术创作和新闻出版工作，推进市县两级有线电视数字化整体转换、数字家庭行动计划和数字电影及农村电影免费放映的"2131"工程，深入开展群众性文化活动，大力发展文化产业，促进文化与经济融合。深入实施"人才强市"战略，积极推进粤西区域性人才市场建设，创新人才管理，促进人才交流与合作，加快高技能人才的培养引进。加快推进公共卫生体系建设，组建全市突发性公共卫生事件监测系统。推进市中医院新院建设，发展中医药事业。建立完善县、镇、村三级农村医疗卫生服务体系和城市社区卫生服务体系，提高城乡医疗服务水平。完善农村合作医疗补助办法，推进新型农村合作医疗制度，力争今年覆盖面达到80%。规范药品和医疗器材集中招标采购和医疗服务项目收费，切实减轻群众看病负担。严密防控艾滋病、狂犬病、结核病等重大传染病及虫媒传播疾病，继续加强职业病防治。加快120急救指挥中心建设。广泛开展群众性体育运动，提高竞技体育水平，发展体育产业。推进市体育场、游泳中心、体育运动学校和全市行政村篮球场建设，办好中国（阳江）国际风筝会暨全国风筝锦标赛和第三届市运动会。加强人口与计划生育工作，稳定低生育水平，促进出生人口性别比趋于平衡。配合省地震局推进地震海啸预警研究基地建设。加强国防教育、民兵预备役、国防动员和"双拥"工作。继续做好民族、宗教、外事、侨务、人防、统计、民政、老龄、残疾人、气象、妇女、儿童、档案、市志等工作。

　　九、切实关注民生，推进和谐社会建设

　　推进城乡统筹就业。实施积极就业政策，大力发展非公有制经济、中小企业，多渠道增加就业岗位。进一步落实新一轮就业再就业扶持政策，促进下岗失业人员实现再就业。健全市、县、镇（街道）三级公共就业服务机构，建立城乡统一的劳动力市场，完善就业援助体系，推进城乡统筹就业。优化农民进城务工经商的政策环境和体制环境，促进农村富余劳动力转移就业。加强职业技能培训，重点抓好下岗失业人员、农村富余劳动力、失地农民和退役士兵的职业技能培训，提高劳动者就业能力。继续抓好高校毕业生就业和复员转业军人安置工作。

　　提高社会保障水平。健全社会保险市级统筹机制，完善养老、失业、工伤、医疗和生育保险制度，抓好非公有制企业、城镇个体工商户和灵活就业人员的参保工作，扩大社会保险覆盖面。加强社保基金的监管，确保基金安全运作。强化社保基金征缴，建立以失地农民为重点的农村社会保险制度，加大农村社会保障的财政投入，提高农民养老保障水平。改革养老金计发办法，逐步做实养老保险个人账户，推行企业年金制。全面落实城乡最低生活保障制度和五保供养制度，实现应保尽保。

　　关心维护群众利益。坚持以人为本、民生优先、心系群众，切实把民生问题摆在更加突出的位置，以解决人民群众最关心、最直接、最现实的利益问题为重点，尽最大努力、千方百计为群众办实事、好事。继续推进农村贫困户泥砖住房改造和饮水工程建设，全面完成镇通建制村道路硬化建设扫尾工作，推进农村客运候车亭建设，坚持开展卫生下乡活动，加强法律援助中心建设。大力整治群众关心的医疗、药品、煤气、化肥、农药、物业管理等热点价费问题，确保关系国计民生的主要商品和服务价格保持稳定。加快征地制度改革，严格贯彻省委张德江书记关于征地建设的"三条红线"：征地手续不齐全、不完备的项目，不能开工；没有与农民就征地补偿民主协商、达成协议的项目，不能开工；征地补偿款没有兑现到农民手里、各种补偿不到位的项目，不能开工；依法保障被征地农民合法权益。认真查处农村征地、城镇房屋拆迁、国企改革等工作中损害群众利益的行为，妥善做好山林权属纠纷的调处工作，维护群众合法权益。推进扶贫开发，扶持山区老区建设。发展残疾人和慈善事业，加强救灾救济，妥善解决农村"五保户"、优抚对象和特殊群众的生活保障等问题。

　　协调社会利益关系。规范企事业单位工资支付行为，严格执行最低工资制度，维护劳动者合法权益。加强劳动保障监察执法和劳动争议仲裁，促进劳动关系和谐。加强和改进信访工作，认真对待和合理解决群众反映的问题。加强社会矛盾排查调处，依法规范信访秩序，预防和妥善处理群体性事件。

　　强化治安综合治理。健全城乡社会治安防控体系，继续保持高压态势，集中打击"双抢"、"两盗"等多发性犯罪，严厉打击黑恶势力和暴力性犯罪等各种刑事犯罪，坚决遏制"黄赌毒"滋生蔓延。强化交通、消防管理，加强流动人口和出租屋监管，重视做好刑释解教人员安置帮教和社区矫正工作。

　　重视抓好应急管理和安全生产。建立健全突发公共事件应急委员会及其办公室，完善突发公共事件应急处置机制，提高应对突发公共事件能力。认真落实安全生产责任制，严格安全生产许可制度，完善各级安全生产监管机构建设，加强安全生产综合监管。建立健全重大危险源登记建档制度和重大安全隐患整改督办制度，加强事故隐患排查整改，促进安全生产形势稳定好转。

　　十、加强政府自身建设，全面提升政府效能

　　加强民主法制建设。自觉接受人大及其常委会的监督，认真落实人大及其常委会的决议、决定，及时向人大及其常委会报告工作。积极支持人民政协履行政治协商、民主监督、参政议政职能，主动听取各民主党派、工商联、无党派人士及各人民团体的意见建议。认真办好人大代表议案、建议和政协委员提案。逐步完善重大事项社会公示制度、社会听证制度和专家咨询制度，推进决策的民主化、科学化、制度化。健全完善政务公开和民主管理制度，推进村民居民自治和企事业单位民主管理。深入开展"五五"普法工作，提高公民的法律素质和社会法治化管理水平。

　　创新行政管理体制。进一步转变政府职能，切实把政府经济管理职能转到主要为市场主体服务和创造良好发展环境上来。继续加强法制、监察、审计等工作，完善权责明确、行为规范、监督有效的行政执法体制，健全过错责任追究制度，提高政府依法行政水平。推进电子政务建设和"一站式"服务，提高服务效能，努力建设为民、高效、廉洁、务实、有为政府。

　　继续改进机关作风。发扬求真务实作风，切实加强调查研究，做到察实情、说实话、务实事、求实效。切实精简会议、文电，集中精力抓落实。发扬艰苦奋斗优良传统，厉行节约，反对浪费。深入开展廉政建设和反腐败斗争，严肃查处违法违纪案件。认真实施《公务员法》，加强公务员队伍建设，提高公务员素质和服务水平。坚持开展行风检查和评议活动，加强效能监察，确保政令畅通，提高政府机关行政效率。

　　各位代表，回顾过去，我们豪情满怀；展望未来，我们信心百倍！让我们紧密团结在以胡锦涛同志为总书记的党中央周围，高举邓小平理论和"三个代表"重要思想伟大旗帜，全面落实科学发展观，按照省委、省政府和市委的决策和工作部署，以奋发有为的精神状态和求真务实的工作作风，开拓进取，扎实工作，为我市与全省同步全面建成小康社会、率先基本实现社会主义现代化而努力奋斗！
